# Reinforcing community health workers program in africa for universal health coverage and global health security: A call for concerted efforts

**DOI:** 10.1371/journal.pgph.0003727

**Published:** 2024-09-17

**Authors:** Ngashi Ngongo, Nebiyu Dereje, Mohamed El Teriaky, Mosoka Papa Fallah, Tamrat Shaweno, Mohammed Abdulaziz, Tajudeen Raji, James M. Guwani, Morenike O. Folayan, Nicaise Ndembi, Jean Kaseya

**Affiliations:** 1 Africa Centres for Disease Control and Prevention, Addis Ababa, Ethiopia; 2 Department of Child Dental Health, Obafemi Awolowo University, Ile-Ife, Nigeria; PLOS: Public Library of Science, UNITED STATES OF AMERICA

A vibrant primary healthcare system is essential to ensure the attainment of Universal Health Coverage (UHC) and the global health security (GHS) agenda. Community health workers (CHWs) are critical elements of a functional primary healthcare system that bridge the community and the healthcare system itself [1, 2]. CHWs are the lowest level of health cadres, mostly women (70%), who are trusted members chosen by the local community members or organizations to provide basic health awareness and services within their community [3, 4]. CHWs often understand their community better as they are often recruited from the community, enabling them to serve as a bridge between health services and the community. The CHWs might be known as health extension workers, village health workers, community health agents, and community health promoters in different countries [4, 5].

Community health workers’ contributions to disease prevention, control, and health promotion have been demonstrated in peaceful times and during public health emergencies such as the COVID-19 pandemic, Ebola outbreak, and HIV epidemic control. During these crises, CHWs played pivotal roles in community education, contact tracing, vaccine distribution, and providing essential health services to underserved populations [6]. Their deep connections within communities enabled them to build trust, disseminate accurate health information, and address misconceptions. Furthermore, CHWs helped to maintain continuity of care by facilitating access to medical services and supporting patients in adhering to treatment regimens [7]. Their efforts not only helped to curb the spread of diseases but also mitigated the impact of the pandemics on healthcare systems, highlighting their critical role in strengthening public health infrastructure and resilience. As demonstrated by the Ethiopian health extension program, the CHWs have substantially contributed to the strengthening of health systems, thereby contributing to meeting health-related targets of the Millennium Development Goals by 2015 and progressing to meeting the Sustainable Development Goals by 2030 [5]. A systematic review by Blanchard et al. reported that CHW interventions can address inequity gaps in maternal and newborn healthcare [8]. A study in Liberia also indicated that the percentage of malaria diagnoses confirmed by microscopy or rapid diagnostic tests increased from 71% to 95% attributed to the CHW program [9].

However, multi-faceted programmatic and operational challenges such as poor structural and functional governance of CHW’s program, siloed and fragmented coordination approaches followed by partners, and resource constraints, have been negatively impacting the effectiveness of the program in Africa and other low-and-middle income countries (LMICs)–underscoring the need for concerted and coordinated efforts from all the stakeholders involving in the CHW’s program [3, 5, 10]. In many African countries, CHWs often lack clear governance and policies–resulting in ambiguities in recruitment, financing, incentivizing, career development, and sustaining of CHWs. Despite various recommendations such as the Monrovia Call to Action [11], many African countries struggle to institutionalize CHWs as their cadre of health workers. Although the number of CHWs in Africa doubled during the COVID-19 pandemic and reached about 0.6 CHWs per 1000 people (Fig 1), this number is less than half of the required 2 million CHWs in the region [12].

**Fig 1:**
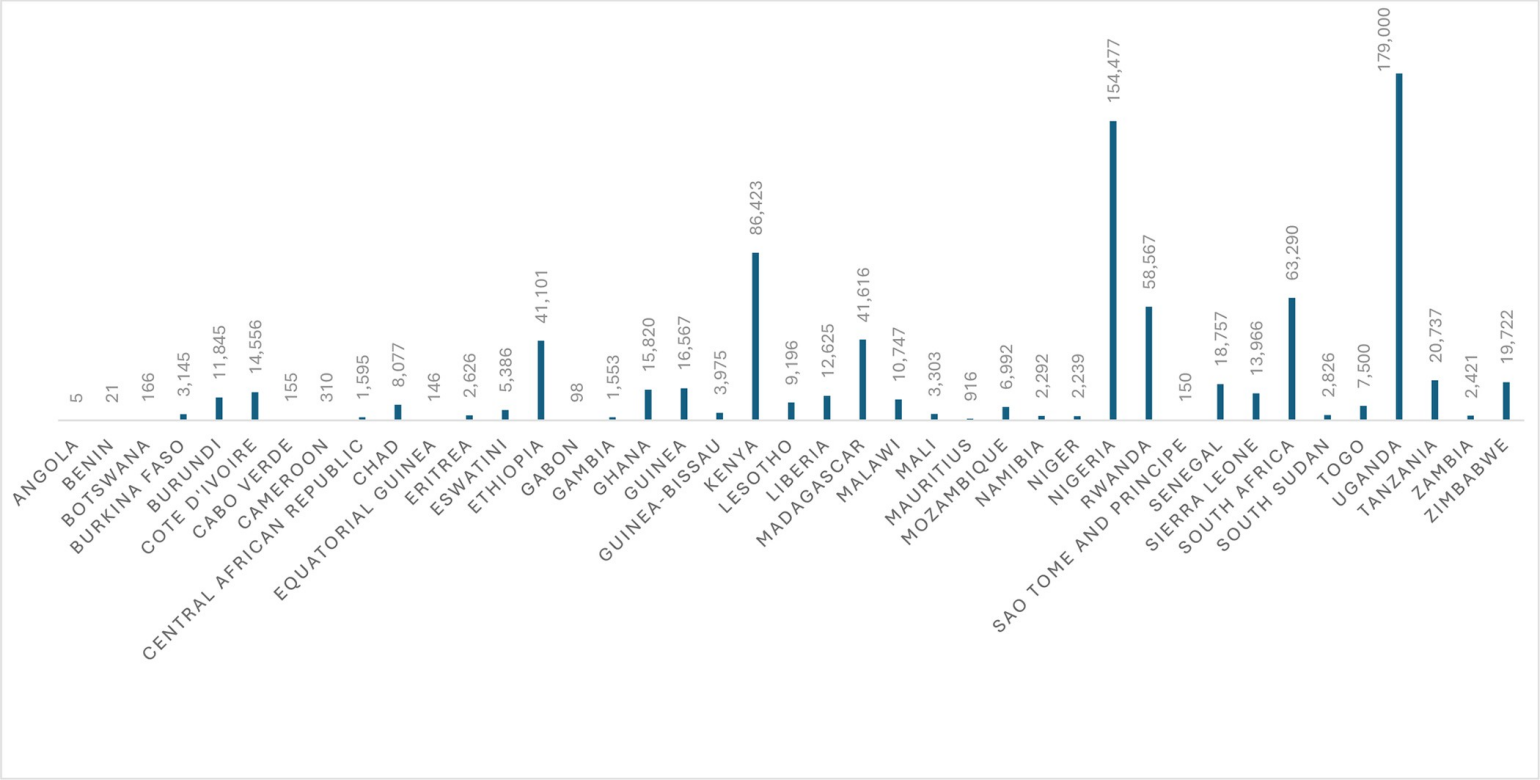
Number of CHWs in Africa–as per the WHO’s latest data.

CHW programs are also underfunded with a $5.4 billion annual funding gap [13], partly because funding systems are largely disease-focused and characterized by siloed and fragmented donor-driven approaches [4, 10]. The government’s lack of political prioritization and insufficient domestic resources for CHW programs also contribute to poor programmatic outcomes. CHW programs have also been affected by operational challenges such as a lack of required supplies and equipment (including personal protective equipment), lack of appropriate training (pre-service, in-service, and refresher training), and sometimes stigma and discrimination from the community, particularly during public health emergencies [5, 14].

## A call for concerted action

Strengthening primary healthcare delivery system requires comprehensive policy development and in-country political prioritization that addresses the integration of CHWs into the formal healthcare systems in Africa–ensuring they are adequately trained, supervised, and compensated [3, 10]. Policies should also focus on improving or strengthening the healthcare referral systems from the communities, securing sustainable funding for the national CHW programs, and fostering partnerships between government, non-governmental organizations, and international bodies to pool human and financial resources for the national CHW programs. Notably, meeting the financial gap for the scalability and sustainability of the CHW program requires global solidarity and shared responsibility.

Funding from various multilateral organizations such as The Global Fund, PEPFAR, UNAIDS, WHO, and other sources can be directed to support CHW programming worldwide. The Pandemic Fund, established to channel critical investments to strengthen pandemic prevention, preparedness and response (PPPR) capacities at national, regional, and global levels, is another great opportunity for funding CHWs. As indicated by various agreements such as the Monrovia Call to Action in March 2023, a call for the adoption of a unified One Plan, One Budget, and One M&E framework at the high-level ministerial event on CHW during the World Health Assembly in May 2023, and the Lusaka Agenda in 2024, harmonization of various funding sources and coordination of activities to support and sustain country-led CHW programs has never been more critical than now. For this reason, Africa CDC launched joint initiatives to support community health programs that resulted in securing a $900 million commitment to community health investments, of which 74% was earmarked for Africa [15].

Learning from the history of contributions made by CHWs, particularly during the COVID-19 pandemic, African governments need to make supporting CHWs a major part of their pandemic preparedness plan and develop policy frameworks to guide its coordinated implementation during pandemics. This prioritization should follow the development of a time-bound strategic plan to sustain and expand the number and scope of CHWs and address operational challenges of such engagement. Such a strategic plan should include clear guidelines for the recruitment, training, and retention of CHWs, as well as providing them with adequate resources and support. Furthermore, the plan should focus on integrating CHWs into the broader healthcare system, ensuring they have access to the necessary tools and technologies to perform their duties effectively. Coordination mechanisms should also be established to enhance collaboration between CHWs, healthcare professionals, and relevant stakeholders. By addressing these operational challenges and fostering a supportive environment, African governments can ensure that CHWs are well-prepared and equipped to respond to future health emergencies.
